# Outcomes with loncastuximab tesirine following CAR T-cell therapy in patients with relapsed or refractory diffuse large B-cell lymphoma

**DOI:** 10.1038/s41408-024-01195-4

**Published:** 2024-11-28

**Authors:** Narendranath Epperla, Melanie Lucero, Tom Bailey, Laura Mirams, Jolenta Cheung, Mona Amet, Gary Milligan, Lei Chen

**Affiliations:** 1 Division of Hematology and Hematologic Malignancies, Huntsman Cancer Institute, University of Utah, Salt Lake City, UT USA; 2 ADC Therapeutics, New Providence, NJ USA; 3 Adelphi Real World, Macclesfield, UK

**Keywords:** Medical research, Health care

## Abstract

The efficacy of loncastuximab tesirine (lonca) following chimeric antigen receptor T-cell therapy (CAR-T) progression/failure is unknown. Hence, we sought to examine real-world use and outcomes of lonca following CAR-T in patients with relapsed or refractory (R/R) diffuse large B-cell lymphoma (DLBCL) in the USA. In this retrospective study, we included adults (age ≥ 18 years) with R/R DLBCL who received lonca monotherapy as third- (3 L) or fourth line (4 L) treatment after progressing on second line (2 L) or 3 L CAR-T, respectively. Post-CAR-T lonca outcomes included response rates (overall response rate [ORR] and complete response [CR] rate), duration of response (DOR), progression-free survival (PFS), and overall survival (OS). A total of 118 patients were included in the analysis with 95 receiving lonca following 2 L CAR-T (median age:66 years; 61% male) and 23 following 3 L CAR-T (median age:57 years; 43% male). Patients with 2 L CAR-T/3 L lonca had an ORR of 73% (CR rate of 34%). With a median follow-up of 8.5 months following lonca initiation, median DOR, PFS, and OS were not reached. The DOR, PFS, and OS at 12 months were 68%, 77%, and 84%, respectively. Patients with 3 L CAR-T/4 L lonca had an ORR of 78% (CR rate of 17%). With a median follow-up of 13 months following lonca initiation, the median DOR and PFS were 7.6 and 12.0 months, while median OS was not reached. OS at 12 months was 95%. In this study, we found that lonca monotherapy was an effective treatment option in R/R DLBCL in 3 L and 4 L settings including those who were resistant to or progressed after CAR-T.

## Introduction

Diffuse large B-cell lymphoma (DLBCL) is the most common non-Hodgkin lymphoma (NHL), accounting for approximately 20–30% of new cases annually [1, 2]. Although DLBCL is often curable, almost 10–15% of patients develop primary refractory disease following standard first-line chemoimmunotherapy [3, 4]. Overall survival (OS) in patients with refractory disease is approximately 6 months [5]. In addition, 20–25% of patients relapse, typically within the first 2 years [6]. For those with early relapse, the 3-year event-free survival rate is only 20%, compared to 40–50% in those who relapse after 12 months or more [7]. Until recently, second line (2 L) treatment options in patients with relapsed/refractory (R/R) DLBCL were limited to salvage chemotherapy followed by autologous hematopoietic cell transplantation (auto-HCT) in those achieving chemosensitivity [8]. However, only about half of R/R DLBCL patients achieve a response to salvage therapy and are eligible for auto-HCT [7, 9, 10]. Furthermore, patients who progress following auto-HCT have dismal outcomes [11]. The advent of chimeric antigen receptor T-cell therapy (CAR-T) has revolutionized the treatment of R/R DLBCL [12]. In October 2017, axicabtagene ciloleucel (axi-cel) became the first CD19-targeted CAR-T approved by the Food and Drug Administration (FDA) as third- (3 L) or subsequent-line (+) therapy for R/R DLBCL based on the results of the ZUMA-1 clinical trial [13, 14]. Findings from the JULIET and TRANSCEND‐NHL‐001 clinical trials resulted in FDA-approval of tisagenlecleucel (tisa-cel) and lisocabtagene maraleucel (liso-cel) as 3 L + CAR-Ts in 2018 and 2021, respectively [15–18]. The ZUMA-7 trial demonstrated the efficacy of axi-cel after prior chemoimmunotherapy, leading to FDA approval as 2 L DLBCL therapy in April 2022 [19, 20]. Approval of liso-cel for 2 L therapy soon followed based on the TRANSFORM and PILOT trial outcomes [21–23]. Although CAR-T is effective, its use is restricted by treatment toxicity, lack of effective bridging therapy, and limited access [12, 24, 25]. Furthermore, in addition to patients with partial or no response to CAR-T, 40–60% of patients who achieve complete remission eventually relapse, indicating the need for effective post-CAR-T therapies [26, 27]. In April 2021, loncastuximab tesirine (lonca), a first-in-class CD19-directed antibody-drug conjugate, was approved for the treatment of R/R DLBCL after ≥2 prior lines of systemic therapy in the United States (USA). FDA approval was based on the pivotal phase 2 LOTIS-2 trial where 145 enrolled patients received lonca monotherapy resulting in an overall response rate (ORR) of 48.3% and a complete response (CR) rate of 24.1% [28]. However, with CAR-T increasingly being used in the 2 L setting, it is important to understand the efficacy of FDA-approved therapies such as lonca following 2 L CAR-T failure and to understand, more in general, its efficacy following CAR-T [29]. Although lonca demonstrated efficacy as a 3 L+ therapy among the post-CAR-T patients in its registration trial (ORR: 42.9%; CR: 21.4%), there was a limited number of post-CAR-T patients (*n* = 14) and none received 2 L CAR-T [6, 30]. Given the paucity of data, this study aimed to examine real-world use and evaluate outcomes of lonca for the treatment of R/R DLBCL when administered following CAR-T in the USA.

## Methods

### Study design and data source

In this observational study, we assessed data from a non-site-based, physician-led, retrospective medical chart review of patients with R/R DLBCL who initiated lonca monotherapy as their 3 L or fourth line (4 L) treatment after progressing on 2 L or 3 L CAR-T. A detailed description of the chart review process is outlined in Table S1. Briefly, medical oncologists, hematologists/hematologist-oncologists who treat DLBCL across the USA were recruited via a national physician panel; physicians were blinded to the study sponsor. For inclusion in the study, consenting physicians were required to have access to the medical records of a minimum of three eligible adult patients with R/R DLBCL from the time of their DLBCL diagnosis up until the time of data extraction or their date of death; patient eligibility is described in the next section. Physicians then extracted eligible patients’ medical chart data according to a pre-specified electronic case report form (eCRF). The collected data included patient demographics, clinical characteristics, treatment history, treatment patterns of lonca, and clinical outcomes associated with lonca. Automated logic checks were built into the eCRF to ensure that logically impossible data were not entered. At the end of each eCRF, physicians were asked to answer three randomly selected questions that they had previously answered; an inconsistent answer to any one of the three questions resulted in the exclusion of the eCRF from the analyses. Throughout data collection, data points with unknown or unclear responses were queried and updated accordingly. All data was transferred to a single electronic database for de-identification and anonymization prior to analysis.

### Patient selection

Patients were required to have a confirmed diagnosis of R/R DLBCL (DLBCL not otherwise specified [NOS], DLBCL arising from low-grade lymphoma, and high-grade B-cell lymphoma), have received a 2 L or 3 L commercially available CAR-T (axi-cel, liso-cel or tisa-cel), and have evidence of DLBCL progression prior to initiating lonca monotherapy as the next treatment (3 L or 4 L). The patient’s lonca initiation date (index date) had to occur after April 1, 2021, and was required to be ≥6 months prior to the data collection date to allow for a minimum 6-month follow-up period following lonca initiation for the evaluation of treatment outcomes (Supplementary Fig. 1). Patients with a date of death occurring after the initiation of lonca treatment but prior to data collection were not required to have a minimum follow-up period duration to be eligible for inclusion in the study. Patients were also required to be aged ≥18 years on the index date. Patients who participated in any interventional clinical trials following their R/R DLBCL diagnosis, received any systemic treatments for other primary tumors, received a polatuzumab- or tafasitamab-containing regimen as first-line treatment, or had lymphoma with active central nervous system involvement at the index date, including leptomeningeal disease, were excluded from the study. Physicians selected from eligible patients for chart review according to a specified randomization process. Briefly, physicians were given a randomly generated letter of the alphabet and asked to select patients with a surname beginning with that letter or, if there were no patients with a suitable surname, the next sequential letter(s) until a qualified patient was identified. If there were multiple patients with surnames beginning with the same letter, physicians were instructed to select patients in alphabetical order according to the first letter of their first name e.g., ‘Adam Peters’ was selected first followed by ‘Ben Patterson’.

### Ethics approval and consent to participate

The study was approved by the Pearl IRB and was conducted in compliance with the Declaration of Helsinki. All extracted data was processed in accordance with the data protection principles as set out in Article 5 of the EU General Data Protection Regulation (GDPR) and the UK GDPR. As this was a retrospective study, informed consent was waived.

### Outcomes and definitions

Baseline characteristics collected in the eCRF included demographic and clinical characteristics, and treatment history prior to lonca monotherapy. Patient demographics were collected at the index date (age, race, ethnic origin, and health insurance type), as well as concomitant conditions. Patient’s initial lymphoma diagnosis, DLBCL type and cell of origin, DLBCL expression, and transformation status were collected at diagnosis. Patient’s International Prognostic Index (IPI) score, Ann Arbor DLBCL staging, and presence of bulky disease were collected at diagnosis and the index date. Treatment history prior to the index date included all treatments received after diagnosis, best response to pre-index treatments, and dates of relapse and progression. Lonca-related treatment patterns and clinical effectiveness outcomes included the number of cycles, best response to lonca monotherapy, ORR, CR, partial response (PR), duration of response (DOR), progression-free survival (PFS), OS, and cause of death. Responses were assessed by treating physicians per Cheson 2007 or Lugano criteria [31]. ORR was measured as the proportion of patients with a best response of CR or PR. DOR was defined as the time from the earliest CR/PR until the first documented disease progression or death due to DLBCL. PFS was defined as the time from the index date until the earliest of disease progression or death. Treatment interruptions or discontinuations due to adverse events (AEs) during lonca treatment were also collected. Subgroup analyses were conducted to assess the response rates to lonca treatment as well as time-to-event endpoint analyses based on presence or absence of bulky disease. Bulky disease was defined as any tumor ≥7.5 cm in its longest dimension.

### Statistical analysis

Descriptive statistics were utilized to summarize baseline characteristics, lonca-related treatment patterns and clinical effectiveness outcomes. Counts and percentages were reported for categorical variables. Continuous variables were summarized using means, standard deviations (SD), medians, and first and third quartiles (Q1, Q3). Time to event analyses were performed by using the Kaplan-Meier method. If disease progression or death had not occurred at the time of data cut-off, patients were censored on their last encounter date. Patients missing data for a specific variable were excluded from the analysis for that variable only. All data analyses were conducted using ‘Stata’ statistical software version 16.0 (StataCorp LLC., College Station, TX, US).

## Results

Among the 17 hematologists/hematologist-oncologists and 7 medical oncologists recruited during the chart review, 11 (46%) were practicing at an academic/teaching hospital or medical center (including specialist cancer centers), 10 (42%) at a community/non-academic hospital or medical center, and 3 (13%) had office-based practices. Of the 118 R/R DLBCL patients included in the chart abstraction, 95 received 2 L CAR-T/3 L lonca therapy and 23 received 3 L CAR-T/4 L lonca therapy. Only two patients had an additional line of therapy after initiating lonca; no patients subsequently received an auto- or allogeneic HCT.

### 2 L CAR-T/3L lonca treatment

#### Patient characteristics

Patient demographics and clinical characteristics at the index date are presented in Table 1. Among the 95 patients with 3 L lonca monotherapy, the initial diagnosis was DLBCL NOS in 44 (46%) patients, while double-hit and triple-hit accounted for 33%. At 3 L lonca monotherapy initiation, mean patient age was 65.7 years (SD: 9.5 years) and 58 (61%) were male. The most frequent health insurance types were Medicare (*n* = 46, 48%), commercial insurance (*n* = 32, 34%), and Medicaid (*n* = 11, 12%). The majority of patients had stage III or IV disease (*n* = 73, 77%), 24 (25%) had bulky disease, and 62 (65%) had an IPI indicating high-intermediate or high-risk disease. Diabetes (36%) and chronic pulmonary disease (22%) were the most common concomitant conditions.

**Table 1:** Patient demographic and clinical characteristics.

Variable	Loncastuximab line of therapy	
	3rd Line *N* = 95 (%)	4th Line *N* = 23 (%)
Age, median (Q1, Q3), years	66 (61, 72)	57 (50, 64)
Male	58 (61%)	10 (43%)
Race		
White	58 (61%)	15 (65%)
African American	31 (33%)	6 (26%)
American Indian or Alaska Native	1 (1%)	0 (0%)
Asian	5 (5%)	2 (9%)
Hispanic/Latin/Spanish origin		
Yes	7 (7%)	3 (13%)
No	86 (91%)	13 (57%)
Unknown	2 (2%)	7 (30%)
Type of DLBCL diagnosis		
De novo DLBCL	44 (46%)	21 (91%)
DLBCL arising from low-grade lymphoma	32 (34%)	1 (4%)
High-grade B-cell lymphoma	19 (20%)	1 (4%)
Cell of origin		
Germinal center B-cell (GCB)	48 (51%)	16 (70%)
Activated B-cell (ABC)	28 (29%)	3 (13%)
Unknown	19 (20%)	4 (17%)
DHL/THL	31 (33%)	3 (13%)
DEL/TEL	36 (37%)	15 (65%)
Ann Arbor Stage		
Stage I	12 (13%)	0 (0%)
Stage II	10 (11%)	3 (13%)
Stage III	24 (25%)	7 (30%)
Stage IV	49 (52%)	13 (57%)
Presence of bulky disease^a^		
Yes	24 (25%)	2 (9%)
No	69 (73%)	21 (91%)
Unknown	2 (2%)	0 (0%)
IPI risk groups		
Low risk	14 (15%)	4 (17%)
Low-intermediate risk	19 (20%)	14 (61%)
High-intermediate risk	20 (21%)	5 (22%)
High risk	42 (44%)	0 (0%)
Comorbidities		
None	29 (31%)	13 (57%)
Diabetes (without complication)	34 (36%)	4 (17%)
Chronic pulmonary disease	21 (22%)	2 (9%)
Peripheral vascular disease	14 (15%)	3 (13%)
Congestive heart failure	13 (14%)	2 (9%)
Diabetes without end organ damage	11 (12%)	1 (4%)
Myocardial infarction	11 (12%)	2 (9%)
Mild liver disease	11 (12%)	2 (9%)
Cerebrovascular disease (except hemiplegia)	9 (9%)	1 (4%)
Ulcer disease	6 (6%)	1 (4%)
Connective tissue disease	2 (2%)	1 (4%)
Second solid tumor (non-metastatic)	4 (4%)	0 (0%)
Moderate or severe liver disease	2 (2%)	0 (0%)
Other	2 (2%)	0 (0%)

Abbreviations: *DHL* double hit lymphoma, *DEL* double expressor lymphoma, *THL* triple hit lymphoma, *TEL* triple expressor lymphoma, *IPI* international prognostic index, *CAR-T* chimeric antigen receptor T-cell therapy, *DLBCL* diffuse large B-cell lymphoma, *Q1* first quartile, 25th percentile, *Q3* third quartile, 75th percentile.

^a^Presence of bulky disease was defined as any tumor ≥7.5 cm in its longest dimension.

#### Prior treatment history including lonca treatment patterns and AEs

The first-line treatments are outlined in Table S2. Second-line CAR-T treatment histories are presented in Table 2. Few patients received bridging therapy to CAR-T (*n* = 12, 13%). Axi-cel was used in 62 patients (65%) and liso-cel in 33 (35%). Twenty-seven (29%) patients were refractory to CAR-T. The median time to progression was 4 months (Q1, Q3: 1.4, 6.5 months). The median time from 2 L CAR-T infusion to the initiation of 3 L lonca was 6.7 months (Q1, Q3: 3.0, 9.2 months). Lonca treatment patterns are presented in Table 3. Due to AEs, 8 (8%) patients discontinued treatment and 7 (7%) had treatment interruptions/drug holidays (AEs listed in Table S3). The most common AEs leading to discontinuation were neutropenia (*n* = 3), thrombocytopenia (*n* = 3), and anemia (*n* = 3), while the most common AE leading to treatment interruption, or a drug holiday was liver dysfunction as evidenced by aspartate aminotransferase (AST) and alanine transaminase (ALT) levels above the institutional upper limit of normal (*n* = 6). The median follow-up duration was 8.5 months.

**Table 2:** CAR-T treatment history prior to loncastuximab treatment.

	Loncastuximab line of therapy	
	3rd Line *N* = 95 (%)	4th Line *N* = 23 (%)
2nd Line CAR-T		
CAR-T product		
Axicabtagene ciloleucel	62 (65%)	–
Lisocabtagene maraleucel	33 (35%)	–
Best response to 2nd line CAR-T		
Complete response	23 (24%)	–
Partial response	44 (46%)	–
Stable disease	11 (12%)	–
Progressive disease	16 (17%)	–
Unknown	1 (1%)	–
3rd Line CAR-T		
CAR-T product		
Axicabtagene ciloleucel	–	18 (78%)
Tisagenlecleucel	–	5 (22%)
Best response to 3rd line CAR-T		
Complete response	–	12 (52%)
Partial response	–	5 (22%)
Stable disease	–	4 (17%)
Progressive disease	–	2 (9%)
Refractory to CAR-T		
No. with response status	94^a^	23
CAR-T responders	67 (71%)	17 (74%)
Refractory to CAR-T	27 (29%)	6 (26%)
Bridging therapy to CAR-T		
Yes	12 (13%)	13 (57%)
No	78 (82%)	7 (30%)
Unknown	5 (5%)	3 (13%)
Time from CAR-T infusion to progression, days		
No. with recorded dates	55	8
Mean (SD)	135.7 (113.3)	198.9 (98.2)
Median (Q1, Q3)	123.0 (42.0, 198.0)	221.5 (150.8, 254.5)
Time from CAR-T infusion to loncastuximab initiation, days		
No. with recorded dates	75	9
Mean (SD)	205.7 (147.8)	266.0 (109.4)
Median (Q1, Q3)	203.0 (92.0, 280.0)	297.0 (215.5, 358.0)

Abbreviations: *CAR-T* chimeric antigen receptor T-cell therapy, *Q1* first quartile, 25th percentile, *Q3* third quartile, 75th percentile, *SD* standard deviation.

^a^CAR-T response was unknown for *n* = 1 in those receiving CAR-T in the 2 L setting.

**Table 3:** Loncastuximab monotherapy treatment patterns.

	Loncastuximab line of therapy	
	3rd Line *N* = 95 (%)	4th Line *N* = 23 (%)
Follow-up time, months		
Mean (SD)	10.3 (5.6)	12.6 (4.5)
Median (Q1, Q3)	8.5 (6.7, 13.6)	12.9 (11.7, 13.9)
Time from diagnosis to loncastuximab initiation, months		
No. with recorded diagnosis date	80	21
Mean (SD)	20.0 (15.4)	47.9 (29.9)
Median (Q1, Q3)	17.1 (12.8, 24.6)	48.0 (17.4, 72.0)
Number of cycles		
Mean (SD)	6.3 (4.5)	7.4 (3.9)
Median (Q1, Q3)	6.0 (3.0, 8.0)	6.0 (6.0, 9.0)

*CAR-T* chimeric antigen receptor T-cell therapy, *Q1* first quartile, 25th percentile, *Q3* third quartile, 75th percentile, *SD* standard deviation.

#### Efficacy measures

**Fig. 1:**
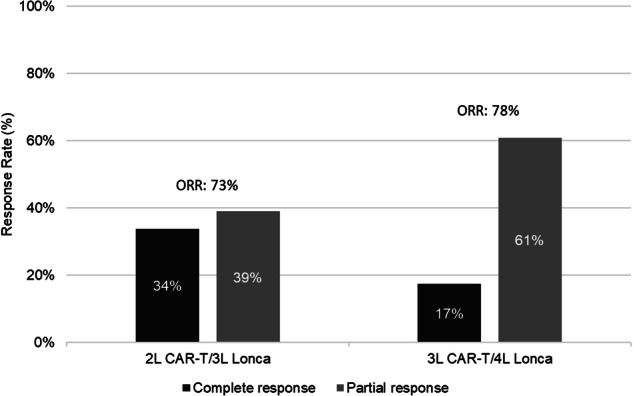
Best Response to loncastuximab monotherapy in 3 L and 4 L setting. Best response to loncastuximab monotherapy in 3L setting following 2L CAR-T and in 4L setting following 3L CAR-T.

Clinical effectiveness measures of lonca monotherapy are presented in Fig. 1 and Table 4. Among patients with 3 L lonca monotherapy, the ORR was 73%; CR and PR rates were 34% and 39%, respectively (Fig. 1). DOR, PFS and OS at 12 months were 67.6%, 76.6% and 83.7%, respectively (Table 4); median DOR, PFS and OS were not reached. Lonca response rates were higher among the 67 patients who had a response to 2 L CAR-T compared to the 27 patients who were refractory to CAR-T (ORR: 82% vs 52%; CR: 43% vs 11%; PR: 39% vs 41%). Among patients who received 2 L axi-cel (*n* = 62) and liso-cel (*n* = 33), 3 L lonca monotherapy produced an ORR of 74% (CR: 32%; PR:42%) and 70% (CR: 36%; PR:33%), respectively. Of the 14 (15%) patients who were deceased at the time of data extraction, 13 had died due to DLBCL, its treatment or associated complications.

**Table 4:** Loncastuximab Monotherapy Outcomes.

	Loncastuximab line of therapy	
	3rd Line *N* = 95 (%)	4th Line *N* = 23 (%)
Overall response rate	69 (73%)	18 (78%)
Best overall response		
Complete response	32 (34%)	4 (17%)
Partial response	37 (39%)	14 (61%)
Stable disease	18 (19%)	3 (13%)
Progressive disease	4 (4%)	2 (9%)
Death	4 (4%)	0 (0%)
Duration of response		
Median (95% CI)	NR (8, NR)	7.6 (6.0, 8.9)
12-month DOR, (%)	68%	22%
Progression-free survival		
Median (95% CI)	NR	12.0 (8.1, 12.0)
12-month PFS, (%)	77%	27%
24-month PFS, (%)	NR	22%
Overall survival		
Median (95% CI)	NR	NR (19.3, NR)
12-month OS, (%)	84%	95%
24-month OS, (%)	79%	64%

*CAR-T* chimeric antigen receptor T-cell therapy, *CI* confidence interval, *NR* not reached, *Q1* first quartile, 25th percentile, *Q3* third quartile, 75th percentile, *SD* standard deviation.

### 3 L CAR-T/4L lonca treatment

#### Patient characteristics

Among the 23 patients with 4 L lonca monotherapy, the initial diagnosis was DLBCL NOS in 21 (91%) patients; double-hit and triple-hit accounted for 13% (Table 1). At 4 L lonca initiation, mean patient age was 57.0 years (SD: 9.6 years) and 13 (57%) were female. The most frequent health insurance types were commercial insurance (*n* = 10, 43%) and Medicare (*n* = 2, 9%); the health insurance coverage of 10 (43%) patients was unknown. Nearly all patients had stage III or IV disease (*n* = 20, 87%), few had bulky disease (*n* = 2, 9%), and 5 (22%) had an IPI indicating high-intermediate or high-risk disease. Diabetes (17%) and peripheral vascular disease (13%) were the most common concomitant conditions.

#### Prior treatment history including lonca treatment patterns and AEs

Bridging therapy to CAR-T was received by 13 patients (57%, Table 2). Axi-cel was the most commonly used CAR-T product (*n* = 18, 78%). More than a quarter of patients were refractory to CAR-T (*n* = 6, 26%) and median time to progression was 7.3 months (Q1, Q3: 5.0, 8.4 months). The median time from 3 L CAR-T infusion to the initiation of 4 L lonca was 11.3 months (Q1, Q3: 7.4, 12.7 months). No patients discontinued treatment, had treatment interruptions, or drug holidays due to an AE. The median follow-up duration was 12.9 months.

#### Efficacy measures

Among patients with 4 L lonca monotherapy, the ORR was 78%; CR and PR rates were 17% and 61%, respectively (Fig. 1). The median DOR was 7.6 months (95% confidence interval [CI]: 6.0, 8.9 months). Median PFS was 12.0 months (95% CI: 8.1, 12.0 months) and PFS at 12 months was 27.4% (Table 4). Median OS was not reached and OS at 12 months was 95.2%. The lonca monotherapy ORR was 82% (CR: 18%; PR: 65%) for patients responsive to 3 L CAR-T (*n* = 17) and 67% for patients who were CAR-T refractory (*n* = 6; CR: 17%; PR: 50%).

Among patients who received 3 L axi-cel (*n* = 18) and tisa-cel (*n* = 5), 4 L lonca monotherapy produced an ORR of 83% (CR: 22%; PR: 61%), and 60% (CR: 0%; PR: 60%), respectively. Of the two patients (9%) who were deceased at the time of data extraction, only one was DLBCL-related.

### Sub-group analysis

Among the recipients of 3 L lonca with available data for bulky disease (*n* = 93), 24 patients had bulky disease and 69 did not. We then compared the outcomes of patients with bulky disease versus non-bulky disease and found that the ORR (75% vs 74%) and CR rates (33 vs 35%) were similar between the two groups. However, the median PFS (11.8 months vs not reached, *p* = 0.003) and 1-year PFS estimate (44% vs 87%) were significantly shorter in patients with bulky disease compared to non-bulky disease (Fig. S2). Similarly, the median DOR (6.2 months vs not reached, *p* = 0.005) and 1-year DOR estimate (31% vs 85%) were significantly shorter in bulky versus non-bulky disease (Fig. S3). While the median OS was not reached for both groups, the 1-year OS estimate was significantly shorter in bulky vs non-bulky disease (65% vs 91%, *p* = 0.03, Fig. S4). Given the small sample size of patients with bulky disease among the recipients of 4 L lonca (*n* = 2), we could not compare the bulky versus non-bulky disease in this setting.

## Discussion

To our knowledge, this is the largest study to date that reports the real-world outcomes of lonca monotherapy following CAR-T failure. This is also the first real-world study that evaluated the lonca outcomes following 2 L CAR-T failure. Among the 95 patients who received 3 L lonca following 2 L CAR-T failure, the response rates were high (ORR of 73%) and durable (12-month DOR of 68%) with a 12-month PFS and OS of 77% and 84%, respectively. Similar findings were noted in those who received 4 L lonca following 3 L CAR-T failure with an ORR of 78% and a median DOR of 7.6 months. The median PFS was 12 months, and median OS was not reached. These findings suggest that lonca monotherapy could be an effective treatment option for patients with R/R DLBCL in 3 L and 4 L settings including those who are resistant to or progressed after CAR-T.

The findings of the LOTIS-2 clinical trial suggested that lonca monotherapy would be an effective treatment option in patients with R/R DLBCL including those who received prior CAR-T (*n* = 14) with an ORR and CR rates of 43% and 21%, respectively [28, 30]. A retrospective study of R/R DLBCL treated with lonca at 21 academic centers that included 112 patients with prior CAR-T therapy, the majority of whom received 4 L+ lonca, reported a post-CAR-T lonca treatment ORR of 31% and CR rate of 15% [32]. At 8 Lymphoma Epidemiology of Outcomes Consortium centers, 20 patients with R/R large B-cell lymphoma who received post-CAR-T 3 L+ lonca treatment reported an ORR of 50% and CR rate of 20% [33]. Finally, a study evaluating the efficacy of anti-CD19 directed immunotherapy after CAR-T among 13 patients who received 3 L to 8 L+ lonca reported an ORR of 36% and CR rate of 18% [34]. In these previous studies, the majority of patients received CAR-T in 3 L+ and had CR rates that are comparable to our study’s response to 4 L lonca following 3 L CAR-T failure. The differences in ORR between our study (78%) and the previous studies could be attributed to variation in the criteria used in the determination of PR. On the other hand, with a CR rate of 34%, this study provides the first real-world evidence that lonca monotherapy could be an effective treatment following 2 L CAR-T failure.

The LOTIS-2 trial reported a median PFS of 1.4 months and a median OS of 8.2 months in the post CAR-T setting (Table S4) [28, 30, 35]. Patients who received post-CAR-T lonca therapy at 21 academic centers had a median PFS and OS of 2.0 and 4.6 months, respectively [32]. The LEO Consortium study of 20 patients who received post-CAR-T lonca reported a median PFS and OS of 3.0 and 4.7 months, respectively [33]. Finally, the 13 patients treated with lonca after CAR-T had a median PFS and OS of 6 and 33.7 weeks, respectively [34]. In part, these differences may be due to the sequencing of CAR-T and lonca therapy, with the presence of additional lines of therapy, either prior to or after CAR-T, impacting treatment efficacy. A study of 514 patients with R/R aggressive B-cell NHL treated with CAR-T reported that a greater number of lines of therapy prior to CAR-T was associated with inferior PFS and OS for all CAR-T patients and inferior PFS in patients experiencing CAR-T failure [36].

Currently, there is no standard of care for the treatment of patients following CAR-T failure (primary or secondary progressors) [37, 38]. There are limited existing real-world evidence, and the findings of most studies are inconclusive due to small sample sizes [27, 36, 39–46]. After CAR-T failure, up to half of patients receive either no treatment or only supportive care [27, 39, 40]. In a study that evaluated treatment patterns among 167 patients who received further treatment after CAR-T failure (e.g., radiation, lenalidomide-based regimens, polatuzumab with bendamustine and rituximab [Polatuzumab-BR], checkpoint inhibitors ± anti-CD20 antibodies, chemotherapy), median PFS and OS were 2.8 and 9 months, respectively, from time of progression post-CAR-T [36]. The highest PFS (5.5 months, *n* = 18) was reported with Polatuzumab-BR treatment (ORR: 72%; CR: 33%). Another study of 50 patients who received salvage therapy after CAR-T failure (e.g., lenalidomide-based regimens, Bruton tyrosine kinase inhibitors, checkpoint inhibitors, chemo-immunotherapy, allogeneic HCT) reported median PFS and OS of 9.7 and 14.4 months, respectively [39]. Epcoritamab and glofitamab are bispecific T-cell engager antibodies recently approved for the treatment of 3 L + DLBCL [47–50]. However, no real-world studies have been reported to date that demonstrate the outcomes associated with bispecific T-cell engager antibodies following CAR-T failure. The findings of the current study suggest that lonca monotherapy is a promising therapeutic strategy for CAR-T failure.

There are limitations inherent to using a retrospective design. Data quality was reliant on accurate and complete reporting by physicians in the eCRF and the availability of complete patient records. However, quality control steps were applied to identify potentially invalid responses and/or respondents, and a pre-specified random process was used by the physicians when selecting eligible patients thereby minimizing potential selection bias. Of note, the median follow-up of the study was relatively short. Additionally, only the AEs that led to lonca treatment interruption or discontinuation were captured rather than all-inclusive AEs. This along with the small sample size of patients receiving lonca in the 4 L setting precluded any evaluation of AEs between 3 L vs 4 L lonca recipients. Lastly, we did not capture CD19 expression at the time of post CAR-T progression/relapse.

## Conclusion

In summary, this is the first real-world study to examine the effectiveness of 3 L lonca following 2 L CAR-T. In this study, we found that lonca monotherapy was an effective treatment option for patients with R/R DLBCL in the 3 L and 4 L setting including those who were resistant to or progressed after CAR-T.

## Supplementary information

Suppl Appendix

## Data Availability

Data is available on reasonable request. The deidentified participant data of this study is available from the corresponding author on reasonable request. Reuse of the data requires permission from all corresponding authors.
