# Supplementary material for: Outcomes with loncastuximab tesirine following CAR T-cell therapy in patients with relapsed or refractory diffuse large B-cell lymphoma
Source: Blood Cancer J. 2024 Nov 28;14(1):210. doi: 10.1038/s41408-024-01195-4 (PMC11604956; doi:10.1038/s41408-024-01195-4)
Supplement: Supplementary file 1 — Suppl Appendix [file 41408_2024_1195_MOESM1_ESM.docx]

**SUPPLEMENTAL APPENDIX**

**Table of Contents** 1

**Study Design:** Figure S1 2

**PFS for bulky vs non-bulky DLBCL patients receiving lonca in 3L setting:** Figure S2 3

**DOR for bulky vs non-bulky DLBCL patients receiving lonca in 3L setting:** Figure S3 4

**OS for bulky vs non-bulky DLBCL patients receiving lonca in 3L setting:** Figure S4 5

**Detailed Chart Review Methodology:** Table S1 6-9

**Prior treatment history:** Table S2 10

**Loncastuximab monotherapy adverse events:** Table S3 11

**Published Post-CAR-T loncastuximab outcomes:** Table S4 12

**Figure S1: Study Design**

To be eligible for inclusion, patients must have received CAR-T in 2L or 3L, and subsequently shown disease progression prior to initiating loncastuximab as 3L/4L treatment (index event), which must occur during the index period.

The follow-up period began at the index date, at least 6 months prior to the data entry date to allow for a minimum follow-up of 6 months.

**FOLLOW-UP PERIOD**

**April 2021**

**≥ 6 months prior to data entry date**

**Date of data entry**

**DATA COLLECTION PERIOD**

**June 2023**

**October 2023**

**INDEX PERIOD**

**Index event**

CAR-T: chimeric antigen receptor T-cell therapy; 2L: second line of therapy; 3L: third line of therapy; 4L: fourth line of therapy.

**Figure S2: Progression-free survival for bulky versus non-bulky DLBCL patients receiving lonca in 3L setting.**

**Figure S3: Duration of response for bulky versus non-bulky DLBCL patients receiving lonca in 3L setting.**

**Figure S4: Overall survival for bulky versus non-bulky DLBCL patients receiving lonca in 3L setting.**

**Table S1: Detailed Chart Review Methodology**

| **Detailed Chart Review Methodology** | |
| --- | --- |
| **Physician Recruitment** | Physicians were recruited by a third-party local fieldwork partner with national coverage. Physicians completed a screener to ensure they met the pre-determined inclusion criteria and were asked to give their informed consent to participate. This was based on their understanding of what is required in terms of completing the eCRFs, the time required and remuneration (in line with fair market value rates).  Physician inclusion criteria:  The following minimal selection criteria were employed to ensure that the sample of treating physicians was appropriate to the disease area and had patient records meeting the study eligibility requirements:   - Qualified as a medical oncologist/hematologist/ hematologist-oncologist. - Be responsible for the treatment and management of adult R/R DLBCL patients. - Have a minimum of n=3 R/R DLBCL patients who meet the eligibility criteria - Must have access to patients’ medical records from the time of DLBCL diagnosis up until the time of data entry (or date of death) providing for a minimum of 6 months follow-up from the index date (or follow-up until date of death if earlier). - Have the date of death recorded in the patient’s medical record for all eligible patients included in the study’s medical chart review where the patient is deceased at or prior to time of data extraction. - Consent to take part in the study.   Physician exclusion criteria:  Physicians who met the following criteria were excluded from this study:   - Do not treat or have access to medical records of eligible R/R DLBCL patients |
| **eCRF** | Upon completion of the screener, physicians were invited to complete an eCRF which should have taken approximately 30 minutes to complete per patient. The eCRF required physicians to provide data to address the study objectives and involved abstracting medical chart data including patient demographics and clinical characteristics, treatment history, treatment patterns, and clinical outcomes. Participating physicians completed and submitted the eCRF securely via an online web portal.  For the purpose of data verification, three data verification questions were asked at the end of each eCRF completed, which consisted of a repeat of some of the questions asked previously in the eCRF. The answers to these questions were verified during the course of fieldwork. Physicians were prompted to amend their responses if the answers provided did not correspond to the answers provided earlier in the eCRF. Further clarification was sought from physicians, via the data collection agency, if the response did not correspond to the answer provided.  Physicians were required to complete a screener for each patient, before abstracting the data into the eCRF, to confirm their eligibility for the retrospective chart review. Upon confirmation of patient eligibility, they were provided with instructions for the patient selection criteria. Physicians were also instructed to refer to the patient’s complete medical record while completing the eCRF and not answer any question from memory. In addition, physicians were given instruction to employ a random selection approach for selecting patient records.  Random selection of eligible patients for inclusion into the patient chart review  A common challenge for retrospective chart reviews is the potential for bias in the selection of records for inclusion in the study. Allowing physicians to freely select records without any controls in place can result in more recent cases being selected, or those which are easier to access (such as those of living patients). In order to mitigate for potential selection bias, physicians were instructed to randomly select from their pool of patients meeting the eligibility criteria, by following the steps below:   - Physicians were asked to provide eCRFs for eligible patients following a random selection process:   - Physicians were given a randomly generated letter of the alphabet and asked if they have any qualifying patients with a surname (last name) beginning with the randomly allocated letter. Physicians then selected all eligible patients with last names starting with this letter (e.g., ‘P’). If the physician did not have an eligible patient whose last name starts with the randomly generated letter, they were asked to select a patient whose last name starts with the next sequential letter working downwards alphabetically (which in this case will be ‘Q’, ‘R’, etc.) until they find a patient who satisfies this requirement. If the physician does not have an eligible patient whose last name starts with the letter ‘Z’, they were asked to select a patient whose last name starts with the letter ‘A’. The physician was then invited to abstract data from the medical record of the selected patient.   - If a physician had multiple patients whose last name begins with a given letter, they were instructed to select patients in alphabetical order in line with the first letter of their first name e.g., ‘Adam Peters’ would be selected first followed by ‘Ben Patterson’.   - If a physician had multiple patients with the same surname, they were instructed to select patients in alphabetical order in line with their first name e.g., ‘Simon Peters’ would be selected first followed by ‘Tom Peters’.   - Physicians were instructed to continue selecting patients who received loncastuximab monotherapy in this way, based on a new randomly generated letter each time.   The limitation of this approach is that given the anonymized nature of the charts provided, it was not possible to validate that physicians had strictly followed the randomized method. However, it was nonetheless the most suitable approach to achieve a random sample for this study.  The eCRF was subjected to pilot testing by 2 physicians who completed a paper format of the eCRF. The same physicians then took part in a qualitative, discursive style follow-up interview (lasting approximately 30 minutes) to allow for unprompted feedback on the materials in detail at a general and individual question level. Physicians were also asked to provide feedback on how difficult it was to find patients to screen for eligibility, and to provide feedback on the instructions for how physicians should select eligible patients.  The data abstracted was required to fall within the pre-defined index window and follow-up periods. Given loncastuximab’s approval in April 2021, April 2021 was used as the start date of the index window. The index date was defined as the date on which the patient initiated loncastuximab monotherapy after progressing on CAR-T therapy within an index window between April 2021 to the date which was 6 months prior to data entry date. Following the index date, there was a minimum follow-up period of 6-months to allow sufficient time to generate clinical effectiveness outcomes. |
| **Data Management** | In the main data collection phase, data was collected via an online eCRF. To ensure the validity of the results, it was necessary to ensure that the data collected was of good quality. This implies minimizing any missing data and ensuring the data collected is accurate. To maximize data quality, the following procedures were undertaken:   - Local fieldwork agencies were thoroughly briefed, detailing study objectives, patient inclusion/exclusion criteria and other logistical aspects of the study. Regular follow-up by telephone/email was carried out to ensure the screening and recruitment was being executed correctly. - The screener and eCRF were carefully designed to contain clear respondent instructions at each question. - Physicians were asked up to 3 validation questions at the end of each eCRF to cross check the answers provided with those provided in the main eCRF. The questions that appeared were randomly selected from a bank of 9 questions that appeared in the main eCRF, for example, patient’s age, smoking status, DLBCL type, cell of origin, date of initial diagnosis (year and month), transformation status, DLBCL stage at index, number of lines of treatment received in total to date, presence of bulky disease, and date of initiation of loncastuximab monotherapy, so that a different selection of questions appeared at the end of each eCRF. Physicians were not allowed to go back to review the answers they previously provided. Any inconsistency in responses were queried with the physician via the data collection agency. If any abstracted data had suspected quality issues, the entire record was removed and replaced with a new record. If the data validation or physician queries resulted in changes to the original abstracted data, all changes were systematically documented in an Excel-based audit log along with the date and rationale for change, as applicable. - On-going quality control checks were performed once in field to ensure data integrity and followed quality control guidance. - The research team reviewed any flagged participants and excluded the respondent data if the data was suspect. - The fieldwork agency was notified that this respondent would need to be replaced and reason for exclusion. - Screening data was checked for each respondent to ensure they were appropriate for the study.   All data abstracted to eCRFs was transferred to a single electronic database. All data was de-identified and anonymized. Missing data was not anticipated, given the data was collected via an online eCRF and each question had to be answered before the participant could proceed. |
| **Quality Control** | A key challenge for any study of this nature was to obtain an accurate, complete eCRF. This was dependent on the correct completion of the study materials and the availability of detailed patient records, which was easier to achieve in a retrospective eCRF study versus a prospective alternative.  Nevertheless, as the study was conducted using a double blinded data collection approach in which the identities of the participants are not disclosed, source data verification was not possible. Instead, a database of results was reviewed and if any queries or inconsistencies in the data needed to be addressed, these were raised with the third-party fieldwork provider. They addressed the concern/query with the physician whilst anonymity of participating physicians was protected throughout. |

**Table S2. Non-CAR-T Treatment History Prior to Loncastuximab Monotherapy**

|  | **Loncastuximab Line of Therapy** | |
| --- | --- | --- |
|  | **3rd Line**  **N=95 (%)** | **4th Line**  **N=23 (%)** |
| **1st line Treatments** |  |  |
| R-CHOP/ R-CHOP like | 63 (66%) | 19 (78%) |
| Intensive induction therapies | 21 (22%) | 2 (0%) |
| BR | 3 (3%) | 0 (0%) |
| R-CVP | 4 (4%) | 1 (4%) |
| R-COMP | 1 (1%) | 1 (4%) |
| R-CEOP | 1 (1%) | 0 (0%) |
| Others | 2 (2%) | 0 (0%) |
| **2nd line treatment** |  |  |
| *Patients with 2nd line treatment, No.* | 0 | 23 |
| *Chemotherapy, No. (%)* |  |  |
| BR | - | 6 (26%) |
| R-ICE | - | 6 (26%) |
| R-DHAP | - | 3 (13%) |
| ICE | - | 2 (9%) |
| Polatuzumab monotherapy | - | 1 (4%) |
| Polatuzumab-B | - | 1 (4%) |
| Polatuzumab-R | - | 1 (4%) |
| R-CVP | - | 1 (4%) |
| R-miniBEAM | - | 1 (4%) |
| Tafasitamab plus lenalidomide | - | 1 (4%) |
| *Stem cell transplant (SCT), No. (%)* | - |  |
| Yes | - | 14 (61%) |
| Autologous SCT | - | 13 (93%) |
| Allogeneic SCT | - | 1 (7%) |
| No | - | 9 (39%) |

BR: rituximab and bendamustine; Polatuzumab-B: polatuzumab, bendamustine; Polatuzumab-R: polatuzumab, rituximab; R-CEOP: rituximab, vincristine, etoposide, cyclophosphamide, prednisolone; R-CHOP: rituximab, cyclophosphamide, doxorubicin, vincristine, prednisolone; R-CVP: rituximab, cyclophosphamide, vincristine, prednisolone; R-DHAP: rituximab with dexamethasone, cytarabine and cisplatin; R-ICE: rituximab with ifosfamide, carboplatin and etoposide; R-miniBEAM: Rituximab with attenuated: carmustine, etoposide, cytarabine, melphalan; R-COMP: rituximab, cyclophosphamide, vincristine, non-pegylated doxorubicin (myocet^TM^) and prednisone.

**Table S3. Loncastuximab Monotherapy Adverse Events***

|  | **Loncastuximab Line of Therapy** | |
| --- | --- | --- |
|  | **3rd Line**  **N=95 (%)** | **4th Line**  **N=23 (%)** |
| **AEs that led to treatment discontinuation** | |  |
| No. with treatment discontinuation | 8 | 0 |
| Neutropenia | 3 (38%) | 0 (0%) |
| Thrombocytopenia | 3 (38%) | 0 (0%) |
| Anemia | 3 (38%) | 0 (0%) |
| Leukopenia | 2 (25%) | 0 (0%) |
| ALT > institutional ULN | 1 (12%) | 0 (0%) |
| AST > institutional ULN | 1 (12%) | 0 (0%) |
| Diarrhea | 1 (12%) | 0 (0%) |
| Infusion reactions | 1 (12%) | 0 (0%) |
| Nausea | 1 (12%) | 0 (0%) |
| Other | 3 (38%) | 0 (0%) |
| **AEs that led to treatment interruption** | | |
| No. with treatment interruption/drug holiday | 7 | 0 |
| AST > institutional ULN | 3 (43%) | 0 (0%) |
| ALT > institutional ULN | 3 (43%) | 0 (0%) |
| Neutropenia | 2 (29%) | 0 (0%) |
| Anemia | 1 (14%) | 0 (0%) |
| Diarrhea | 2 (29%) | 0 (0%) |
| Nausea | 2 (29%) | 0 (0%) |
| Edema | 1 (14%) | 0 (0%) |
| Infection requiring hospitalization | 1 (14%) | 0 (0%) |
| Leukopenia | 1 (14%) | 0 (0%) |
| Lymphopenia | 1 (14%) | 0 (0%) |
| Rash | 1 (14%) | 0 (0%) |
| Thrombocytopenia | 1 (14%) | 0 (0%) |
| Vomiting | 1 (14%) | 0 (0%) |

ALT: alanine transaminase; AST: Aspartate aminotransferase; ULN: upper limit of normal

* Only the AEs that led to loncastuximab tesirine treatment interruption or discontinuation were captured rather than all-inclusive AEs

**Table S4. Published Post-CAR-T Loncastuximab Treatment Outcomes ^a^**

|  | **Caimi et al^1,2,3^** | **Ayers, et al^4^** | **Nastoupil, et al^5^** | **Iqbal, et al^6^** |
| --- | --- | --- | --- | --- |
|  | n=14 | n=112 | n=20 | n=13 |
| Study type | Clinical trial | RWE study | RWE study | RWE study |
| Loncastuximab line of therapy | 3-8+ | 2-12 | 3+ | 3-8+ |
| Overall response rate | 43% | 31% | 50% | 36% |
| Complete response | 21% | 15% | 20% | 18% |
| Partial response | 22% | 16% | 30% | 18% |
| Duration of response, median (95% CI), months | 8.0 (3.4, NR) | - | 4.0 | 2.7 (0.7, 4.5) |
| Progression-free survival, median (95% CI), months | 1.4 (0.7, NR) | 2.0 (1.6, 2.7) | 3.0 (2.0, 9.3) | 1.4 (0.9, NR) |
| Overall survival, median (95% CI), months | 8.2 (4.7, NR) | 4.6 (3.2, 6.1) | 4.7 (3.7, NR) | 7.8 (2.1, NR) |
| Overall survival at 12 months, % | 33.3% | - | - | - |

CI: confidence intervals; NR: not reached; RWE: real-world evidence

^a^ These data are provided for informational purposes only and are not meant to be compared side to side.

**References**

1. Caimi PF, Ai W, Alderuccio JP, et al. Loncastuximab tesirine in relapsed or refractory diffuse large B-cell lymphoma (LOTIS-2): a multicentre, open-label, single-arm, phase 2 trial. *Lancet Oncol.* 2021;22(6):790-800.

2. Caimi PF, Ardeshna KM, Reid E, et al. The AntiCD19 Antibody Drug Immunoconjugate Loncastuximab Achieves Responses in DLBCL Relapsing After AntiCD19 CAR-T Cell Therapy. *Clin Lymphoma Myeloma Leuk.* 2022;22(5):e335-e339.

3. Caimi PF, Ai WZ, Alderuccio JP, et al. Loncastuximab tesirine in relapsed/refractory diffuse large B-cell lymphoma: long-term efficacy and safety from the phase II LOTIS-2 study. Haematologica. 2024 Apr 1;109(4):1184-1193

4. Ayers E, Zelikson V, Gurumurthi A, et al. Loncastuximab in High-Risk and Heavily-Pretreated Relapsed/Refractory Diffuse Large B-Cell Lymphoma: A Real World Analysis from 21 US Centers. *Blood.* 2023;142:312.

5. Nastoupil LJ, Andersen CR, Ayers A, et al. Effectiveness of Chemo-Immunotherapy (CIT) and Novel Therapies in Second or Later Line of Therapy (2L+) for Patients with Relapsed/Refractory (R/R) Aggressive Large B-Cell Lymphoma (LBCL). *Blood.* 2023;142:309.

6. Iqbal M, Jagadeesh D, Chavez J, et al. Efficacy of CD19 directed therapies in patients with relapsed or refractory large b-cell lymphoma relapsing after CD19 directed chimeric antigen receptor T-cell therapy. *Bone Marrow Transplant.* 2023.
